# Acute spontaneous subdural hematoma as an inaugural presentation of systemic lupus erythematosus with acquired factor XIII deficiency: a case report

**DOI:** 10.11604/pamj.2021.39.207.26336

**Published:** 2021-07-21

**Authors:** Tamba Marc Sandouno, Houda Bachir, Habiba Bennesser Alaoui, Siham Hamaz, Ahmed Amine Eloumri, Mohammed Berrimi, Khalid Serraj

**Affiliations:** 1 Internal Medicine, Immunohematology and Cellular Therapy Laboratory, Medical School of Medicine Oujda, University Mohammed I, Oujda, Morocco

**Keywords:** Spontaneous, acute subdural hematoma, factor XIII, systemic lupus erythematosus, case report

## Abstract

Acute spontaneous subdural hematoma is a rare clinical situation. Among its various etiologies, underlying coagulopathy is associated with a considerable risk of mortality. A 43-year-old female patient with no comorbidity and no personal or family history of bleeding disorders, consulted for acute and intense headache. The brain computed tomography (CT) scan showed a compressive left fronto-parietal acute subdural hematoma. The cerebral magnetic resonance angiography and routine hemostasis workup were normal. Factor XIII activity was low at 41% and the etiological investigation was consistent with the diagnosis of systemic lupus erythematosus. Surgical evacuation of the hematoma, factor XIII supplementation and systemic corticosteroid therapy with hydroxy chloroquine resulted in a favorable outcome. Acquired factor XIII deficiency should be systematically investigated for any acute spontaneous subdural hematoma with a normal hemostasis assessment in an adult with no personal or family history of hemorrhage.

## Introduction

Acute spontaneous subdural hematoma (ASSH) is a relatively rare event. Headache of rapidly increasing intensity is the usual mode of presentation, but the clinical course can be rapidly fatal if not managed promptly. Its various etiologies include, among others, acquired or constitutional disorders of hemostasis [1]. Factor XIII deficiency is a rare coagulation disorder often responsible for hemorrhagic manifestations whose prognosis is largely dominated by intracranial bleeding, which is intraparenchymal in more than 90% of cases [2]. It may be acquired in a variety of situations, including autoimmune diseases, mainly represented by systemic lupus erythematosus (SLE) [3]. Clinical cases with various combinations of SLE, factor XIII deficiency or ASSH have been reported in the literature; however, while in most of the cases described in the literature the diagnosis of SLE was previously known or the bleeding manifestations essentially extra-neurological, this observation reports, to the best of our knowledge, the first case of ASSH as an inaugural presentation of SLE complicated by factor XIII deficiency. It also highlights some limitations in the diagnostic and therapeutic management of acquired factor XIII deficiency in a resource-limited country.

## Patient and observation

**Figure 1:**
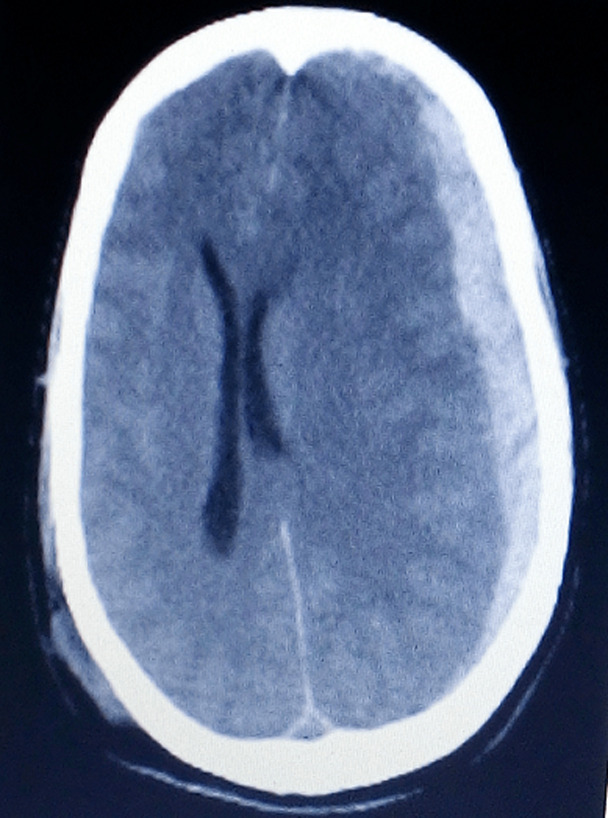
axial non contrast cerebral CT scan showing an acute fronto-parietal left subdural hematoma responsible for a mass effect on the left cerebral hemisphere and subfalcorial engagement

**Figure 2:**
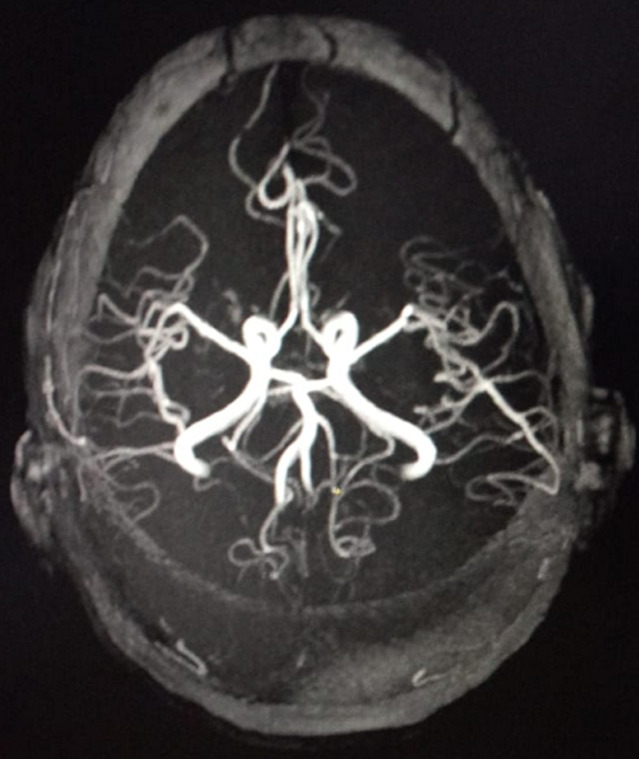
cerebral magnetic resonance angiography showing no arteriovenous malformations

**Figure 3:**
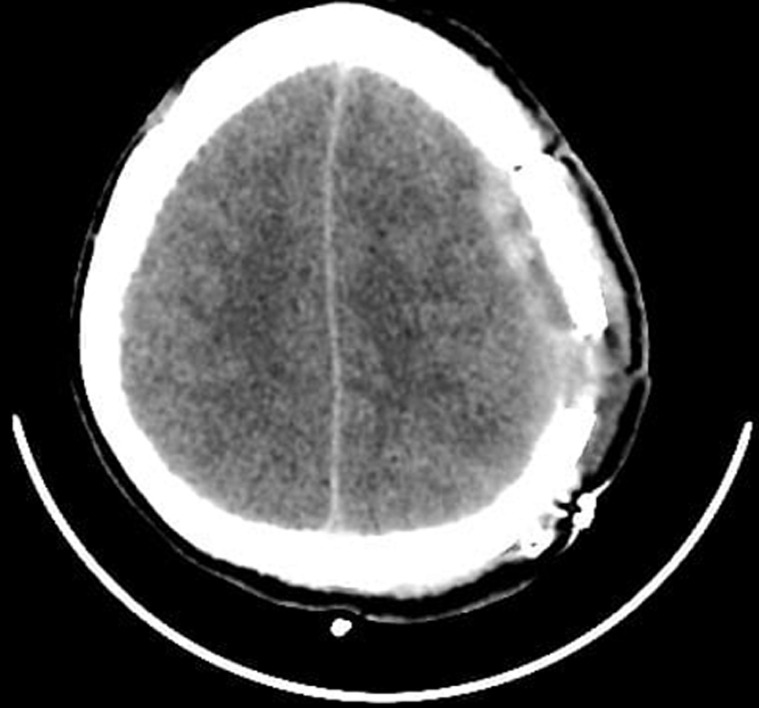
post-operative axial non contrast cerebral CT scan showing surgical stigmas and disappearance of acute subdural hematoma

A 43-year-old female patient with no significant medical history consulted for a headache of abrupt onset and rapidly increasing intensity and resistant to analgesics. She was not known to be hypertensive or addicted to drugs and had no personal or family history of bleeding disorders. She was conscious on admission and had no signs of focalization or meningeal syndrome. Her blood pressure, heart rate and temperature were respectively of 140/80 mmHg, 120 beats/min and 37°C. Non contrast cerebral CT scan revealed a compressive left fronto-parietal subdural hematoma (Figure 1). Routine hemostasis tests showed a platelet count of 130 giga/l, a prothrombin level of 70% and an activated partial thromboplastin time (aPTT) of 12 seconds. Surgical evacuation had been performed but the success of the procedure was short-lived as the hematoma had rapidly reconstructed to indicate neurosurgical re-intervention under massive infusion of fresh frozen plasma associated with repeated injections of tranexamic acid. In addition, cerebral magnetic resonance angiography did not show any underlying arteriovenous abnormalities (Figure 2). We maintained daily infusions of fresh frozen plasma (600 mL/d) and tranexamic acid and the post-operative clinical course was satisfactory (Figure 3).

**Figure 4:**
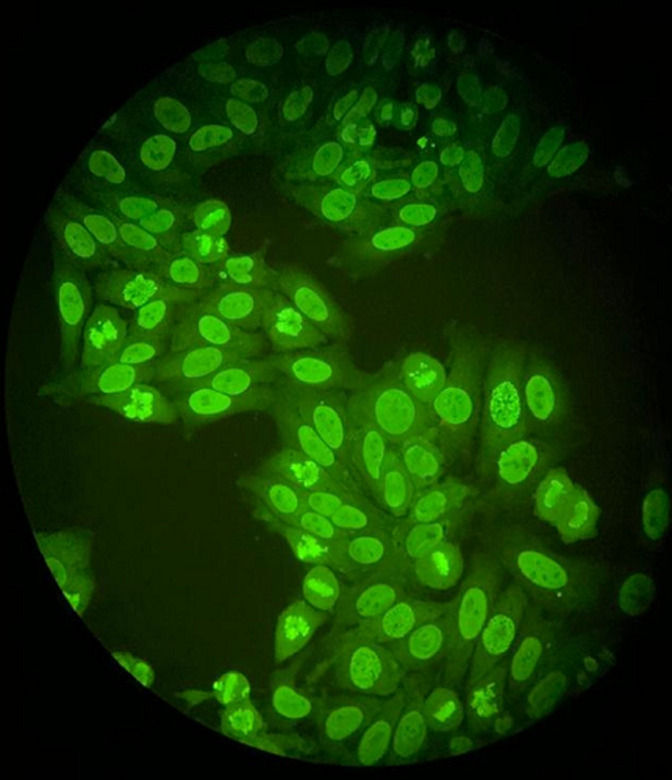
antinuclear antibodies of homogeneous fluorescence (titre=1/1280)

The spontaneous nature of the subdural hematoma associated with a normal hemostasis assessment suggested the hypothesis of an underlying “occult” coagulopathy. We measured factor XIII activity by photometric method and found a low rate of 41% (normal value: 70-140); the biological assessment showed a non-regenerative normochrome normocytic anemia at 7 g/dl, leuko-neutropenia at 3.6 giga/l without thrombocytopenia, C3/C4 hypocomplementemia and polyclonal hypergammaglobulinemia. Bone marrow biopsy and thoraco-abdomino-pelvic CT scan were unremarkable. Antinuclear antibody testing was positive at a rate of 1/1280 with specificity for anti-dsDNA, anti-SSA/Ro and anti-SSB/La (Figure 4). The antiphospholipid antibody test was negative. Liver function, renal function and 24-hour proteinuria were normal.

All clinical and immunobiological findings were consistent with the diagnosis of systemic lupus erythematosus (SLE) with hematologic tropism. Biopsy of the accessory salivary glands was discussed, looking for Gougerot syndrome associated with SLE. However, due to the hemorrhagic risk associated, this invasive procedure was not performed. The discovery of SLE had raised the possibility of an anti-factor XIII autoantibody, but this had not been sought in our context. The patient received an initial bolus of methylprednisolone 15 mg/kg/day for 3 days followed by oral relay at a dose of 1 mg/kg/day of prednisone combined with hydroxy chloroquine 200 mg twice daily at patient´s discharge. She was seen 1 month later and then every 3 to 6 months. The treatment was well observed with good tolerance. Three years later, the patient is free of treatement with no recurrence of hemorrhage, a normal blood count and a factor XIII level of 143%.

## Discussion

Acute spontaneous subdural hematoma (ASSH) is a rare clinical situation. Since it was first described by Munro in 1934, only 193 cases have been reported in a literature review published in 2014. Arterial vascular causes (61.5%), idiopathic forms (10.8%) and coagulation disorders (10.1%) were the main etiologies [1]. Rupture of intracranial aneurysm is classically associated with subarachnoid hemorrhage, but in exceptional situations, ASSH may be a sign of a ruptured aneurysm [4]. The prognosis is then most often pejorative due to diagnostic delay on the one hand or misdiagnosis on the other. This exceptional pathogenic situation with potentially dramatic consequences argues in favor of angio-imaging for all ASSH [4]. It is for this purpose that we performed cerebral magnetic resonance angiography in our context, making it possible to formally exclude the existence of arteriovenous malformation or intracranial aneurysm.

When faced with a spontaneous hemorrhagic event, the search for an underlying coagulopathy is a common practice reflex made easier by the existence of certain risk situations. In the absence of obvious risk factors, a detailed personal or family bleeding history is a valuable and essential step in the diagnostic referral. Global hemostasis tests (prothrombin time, activated partial thromboplastin time, platelet count), carried out as first-line tests, may reveal a primary hemostasis or coagulation disorder. However, these tests are falsely reassuring in factor XIII deficiency [5]. Their constant normality and the relative frequency of intracranial hemorrhage in factor XIII deficiency have motivated the exploration of the latter as a priority in our context. The *International Society on Thrombosis and Hemostasis* (ISTH) have made recommendations for the diagnosis of factor XIII deficiency [6]. The clinical context and certain additional tests may help to distinguish between congenital, acquired, immunological and non-immunological origin. The immunological mechanism is underpinned by the existence of a neutralizing or non-neutralizing autoantibody directed against factor XIII [3].

The frequency of associated autoimmune diseases is estimated to be between 17% and 25% in large published case series with a clear representation of SLE (72% of cases). Analysis of these cases shows the following: the diagnosis of SLE often precedes the diagnosis of coagulopathy with a variable delay of up to several years; compared to other etiologies, patients were younger in the case of SLE-associated deficiency; the hemorrhagic manifestations were essentially extra-neurological [7-9]. In view of the above, we can therefore state that ASSH as the inaugural presentation of SLE with acquired factor XIII deficiency remains an exceptional situation, constituting a real diagnostic and therapeutic challenge. Indeed, how can one evoke factor XIII deficiency in a young adult patient who was previously healthy and without any personal or family medical history? How can SLE be evoked only in the presence of one of its rarest neurological manifestations without any systemic extra neurological manifestation? Young age, female gender, lack of co-morbidity and lack of personal and family history of bleeding have suggested an underlying autoimmunity. The positivity of high titer antinuclear antibodies with fairly specific typing, associated with hypocomplementemia and hypergammaglobulinemia, supported the hypothesis of a SLE with pure hematological tropism. For technical reasons specific to our context, we were unable to demonstrate the existence of anti-factor XIII autoantibodies. However, this in no way calls into question the autoimmune mechanism, which remains possible in accordance with International Society on Thrombosis and Haemostasis (ISTH) recommendations [3] and given the spectacular response under corticosteroid therapy.

Therapeutic management has two components with a dual objective: the first is symptomatic and aims to control bleeding by compensating for the deficiency with exogenous supplies of factor XIII; the second is curative and aims to eradicate the inhibitor causing the deficiency [3]. Various therapeutic means have been shown to be effective in this later goal: corticosteroid therapy, cyclophosphamide, plasmapheresis, intravenous immunoglobulins and rituximab. However, corticosteroid therapy and/or cyclophosphamide are the main therapies used in the majority of reported cases [3,8]. Furthermore, it seems reasonable to combine immunosuppressive therapy with a therapy adapted to the underlying causal pathology. In about 50-68% of cases, immunosuppressive therapy can be stopped without recurrence of bleeding, as was the case in our patient.

In addition to some of the characteristics it shares with the previously mentioned cases, our case presents the following particularities: an inaugural and immediately life-threatening presentation. The occurrence of a severe hemorrhagic syndrome despite a residual factor XIII activity rate of 41% compared to 8.5% on average in the literature; which in the context of an acquired deficit, emphasizes the absence of a critical activity threshold leading to a hemorrhagic manifestation [3,7]. A favourable evolution despite the severity of the clinical picture and the reported highest mortality in the case of underlying coagulopathy (55.6% versus 33.3%) [10]. Finally, in the lack of factor XIII concentrates in our context, the therapeutic strategy combining fresh frozen plasma, tranexamic acid and corticosteroid therapy had been effective. In our opinion, this therapeutic response could be explained by the relatively lower residual level of factor XIII (41%).

## Conclusion

Acquired factor XIII deficiency should be systematically investigated for any acute spontaneous subdural hematoma with a normal hemostasis assessment in an adult with no personal or family history of haemorrhage. If diagnosed early, lupus induced factor XIII deficiency responds to substitution and corticosteroid therapy allowing a favourable outcome.
